# Efficacy of early immunomodulator therapy on the outcomes of Crohn’s disease

**DOI:** 10.1186/1471-230X-14-85

**Published:** 2014-05-03

**Authors:** Min Seob Kwak, Duk Hwan Kim, Soo Jung Park, Tae Il Kim, Sung Pil Hong, Won Ho Kim, Jae Hee Cheon

**Affiliations:** 1 Department of Internal Medicine, Graduate School, Yonsei University College of Medicine, Seoul, Korea; 2 Department of Internal Medicine and Institute of Gastroenterology, Yonsei University College of Medicine, Seoul, Korea; 3 Department of Internal Medicine, Yonsei University College of Medicine, 50 Yonsei-ro, Seodaemun-gu, Seoul 120-752, Korea

**Keywords:** Crohn’s disease, Azathioprine, 6-mercaptopurine, Immunomodulator

## Abstract

**Background:**

The natural course of Crohn’s disease (CD), with continuing relapses and remissions, leads to irreversible intestinal damage. Early adoption of immunomodulator therapy has been proposed in order to address this; however, it is still uncertain whether early immunomodulator therapy could affect the natural course of the disease in real practice. We evaluated the efficacy of such therapy on the prognosis of newly diagnosed patients with CD.

**Methods:**

This retrospective study included 168 patients who were newly diagnosed with CD and who started treatment at Severance Hospital, Seoul, Korea between January 2006 and March 2013. The short- and long-term outcomes were compared between patients treated with early immunomodulator therapy and those treated with conventional therapy.

**Results:**

A Kaplan-Meier analysis identified that administration of immunomodulators within 6 months after diagnosis of CD was superior to conventional therapy in terms of clinical remission and corticosteroid-free remission rates (*P*=0.043 and *P*=0.035). However, *P*=0.827). Patients with a baseline elevated CRP level were more likely to relapse (P<0.005). Drug-related adverse events were more frequent in the early immunomodulator therapy group than in the conventional therapy group *P*=0.029).

**Conclusions:**

Early immunomodulator therapy was more effective than conventional therapy in inducing remission, but not in preventing relapse. Baseline high CRP level was a significant indicator of relapse.

## Background

Crohn’s disease (CD) is a chronic inflammatory disease with unknown etiology that can affect any part of the gastrointestinal tract. Patients with CD have a host of symptoms, including diarrhea, hematochezia, abdominal pain, weight loss, and fever. The last few decades have seen a gradual increase in the number of drugs available for use in the treatment of CD. From a time when only sulfasalazine/5-aminosalicylic acids, corticosteroids, and antibiotics were used, we now have immunomodulators such as thiopurines and methotrexate, as well as biological agents [1]. A step-up strategy, i.e., a progressively intensified method of treatment, is being recommended in current guidelines for medical therapy of CD [2,3].

For mild disease, less toxic but often less efficacious drugs are recommended, while on the other hand, more efficacious yet potentially more toxic drugs are typically administered to patients with severe disease or those who are not responsive to first-line therapy. The purpose of this strategy is to ensure therapeutic endpoints such as induction and maintenance of clinical relief, withdrawal from steroids, and prevention of post-operative relapse. However, conventional treatment has not been successful in reducing complications or the need for surgery [4].

According to several recent studies, more aggressive treatment early in the disease may result in betterresponse and remission rates [2,5,6]. However, one cohort study showed that although immunomodulators have been used more frequently over the last 25 years, there was no significant decrease in the need for surgery in patients with CD [4]. Therefore, it remains uncertain whether early immunomodulator therapy could affect the natural course of disease in real practice. Few studies have focused on the effect of early immunomodulator therapy on the natural course of CD. Accordingly, we evaluated the efficacy of early immunomodulator therapy on the prognosis of patients newly diagnosed with CD in a clinical setting.

## Methods

### Patients

We enrolled a total of 168 patients who were newly diagnosed with CD and who started treatment at Severance Hospital, Seoul, Korea between January 2006 and March 2013. The diagnosis of CD was made according to previously established international criteria based on clinical, endoscopic, histopathological, and radiological findings [7]. The disease extent was determined through endoscopic and/or radiological work-up. Patients who were diagnosed with or suspected to have indeterminate colitis, coexistence of infectious or ischemic colitis, coexistence of other localized or systemic infections, any malignant disease, major systemic illness, connective tissue disease, or inflammatory arthritis were excluded. Patients in the early therapy arm initiated immunomodulator therapy (azathioprine or 6-mercaptopurine) within 6 months of diagnosis. The conventional therapy arm was comprised of patients with CD who initiated immunomodulatory therapy more than 6 months after being diagnosed, or who did not receive immunomodulators during the course of their disease. The Institutional Review Board of the Severance Hospital approved this study.

The Crohn’s Disease Activity Index (CDAI) [8], erythrocyte sedimentation rate (ESR, normal value <15 mm⁄hr), C-reactive protein level (CRP, normal value < 8 mg⁄L), and hematocrit level (Hct, normal value:40.4-52%) were regularly monitored during the follow-up period.

### Thiopurine dosing and follow-up protocol

The initial azathioprine dose (0.5-1.0 mg/kg) was increased to 2.0 to 2.5 mg/kg over one- to four-week intervals unless there were adverse effects. The initial 6-mercaptopurine (6-MP) dose (0.25-0.5 mg/kg) was increased in the same fashion to 1.0 to 1.5 mg/kg. During AZA/6-MP therapy, 5-ASA was administered at a conventional dose (mesalazine 3.0 g/day). Outpatient visits were scheduled at one- to two-week intervals for the first month, a visit at two months, and visits every 2–3 months thereafter, according to the patient’s clinical condition.

### Definition of treatment response and disease relapse

Clinical remission was defined as a CDAI < 150 and clinical response was defined by a 100 point decrease from the baseline CDAI score [9]. Steroid-free remission was defined as maintaining remission for up to 4 weeks after complete withdrawal of corticosteroids. The definition of disease relapse was a CDAI >150 points plus a 100-point increase from CDAI baseline [9].

### Primary and secondary outcome measures

The primary outcome measure was the proportion of patients responsive to treatment at each time point during a 12-month period. The following secondary outcome measures were considered: (1) clinical remission rate of each group, (2) steroid-free clinical remission rate of each group, (3) disease relapse rate of each group, (4) disease-related hospitalization rate of each group, and (5) surgical resection rate of each group.

### Statistics

Statistical analyses were performed with SPSS software (version 18.0 for Windows; SPSS, Inc., Chicago, IL, USA). Continuous variables were compared using a two-tailed Student’s t-test and categorical data were compared using a two-tailed χ2 test or Fisher’ exact test. The clinical response and disease relapse rates after treatment were illustrated using Kaplan Meier survival curves for the grouped factors over the intervention period. Moreover, we analyzed factors associated with relapse by logistic regression analysis. *P*-values <0.05 were considered to indicate statistical significance.

## Results

### Baseline patient characteristics

The baseline characteristics of the 168 patients with CD are listed in Table 1. Of them, 102 patients (81 males, median age ± SD, and 26.5 ± 11.0 years) were treated with early immunomodulator therapy and the other 66 patients (57 males, median age ± SD, 30.0 ± 12.3 years) were treated with conventional therapy. The baseline characteristics of the patients such as age, sex, location of disease, disease behavior, and laboratory findings except for medications used were not significantly different between the two groups.

**Table 1:** Clinical characteristics of patients with Crohn’s disease (N = 168)

**Characteristics**		**Early immuno-modulator therapy**	**Conventional therapy**	***P*-value**
Age, yr, median (IQR)		26.5 (11.0)	30 (12.3)	0.137
Sex, (%)				0.305
	Male	81 (79.4)	57 (86.4)	
	Female	21 (20.6)	9 (13.6)	
Activity status at start medication, (%)				0.474
	Remission	7 (6.9)	5 (7.6)	
	Mild	24 (23.5)	17 (25.8)	
	Moderate	70 (68.6)	41 (62.1)	
	Severe	1 (1.0)	3 (4.5)	
Location, (%)				0.191
	L1 (ileal)	9 (8.8)	2 (3.0)	
	L2 (colonic)	13 (12.7)	7 (10.6)	
	L3 (ileocolonic)	16 (15.7)	13 (19.7)	
	L4 (only upper GI)	0 (0)	3 (4.5)	
	NA	64 (62.8)	41 (62.2)	
Behavior at diagnosis, (%)				0.651
	B1 (non-stricturing, non-penetrating)	20 (19.6)	14 (21.2)	
	B2 (stricturing)	10 (9.8)	3 (4.6)	
	Perianal disease	8 (7.8)	8 (12.1)	
	NA	64 (62.8)	41 (62.1)	
Initial medication, (%)				<0.001
	Only 5-ASA	54 (52.9)	56 (84.8)	
	5-ASA with steroids	9 (8.8)	10 (15.2)	
	Only immunomodulators	3 (2.9)	0 (0)	
	Immunomodulators with 5-ASA	36 (35.3)	0 (0)	
CDAI, median (IQR)		263.0 (119.5)	249.2 (117.9)	0.643
BMI, median (IQR)		19.8 (4.7)	18.9 (3.7)	0.141
CRP, median (IQR)		8.1 (39.2)	8.9 (24.8)	0.052
ESR, median (IQR)		54.0 (59.0)	55.0 (52.0)	0.462
Hct, median (IQR)		38.4 (8.6)	37.3 (7.2)	0.905

IQR, inter-quartile range; CDAI, Crohn’s disease activity index; BMI, body mass index; CRP, C-reactive protein; ESR, erythrocyte sedimentation rate; Hct, hematocrit; NA, not applicable.

### Primary and secondary outcome measures

**Figure 1:**
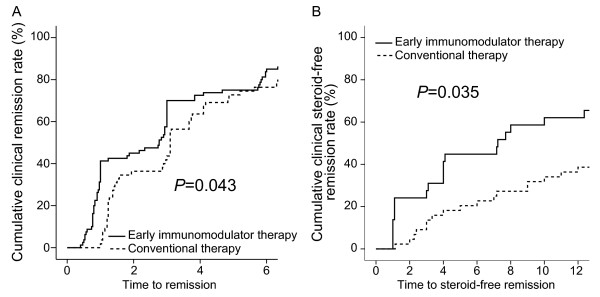
The cumulative probabilities of patients who achieved clinical remission (A), and steroid-free remission (B) between early immunomodulator therapy and conventional therapy groups.

The median length of follow-up for patients was 27 months (Range, 35 or IQR, 21). The clinical remission rates at 6 months were 85.0% in the early immunomodulator group and 76.4% in the conventional therapy group, respectively (Figure 1A). There was a statistically significant difference in the overall remission rate between the two groups (Log Rank test *P* =0.043). In addition, corticosteroid-free clinical remission rates at 6 and 12 months were noted in 44.8% and 62.1% of patients in the early immunomodulator group, respectively, and in 22.7% and 38.6% in the conventional group. This also reached statistical significance (Log Rank test *P* =0.035) (Figure 1B).

**Figure 2:**
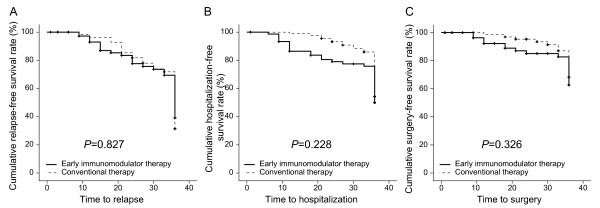
The cumulative probabilities avoiding disease relapse (A), hospitalization (B), and surgery (C) between early immunomodulator therapy and conventional therapy groups.

In the patients treated with early immunomodulator therapy, 7.0% relapsed at 12 months. This percentage increased to 22.3% at 24 months and to 60.9% at 36 months (Figure 2A). In patients treated with conventional therapy the relapse rates at months 12, 24, and 36 were 3.6%, 18.2%, and 68.6%, respectively. There was no statistical significance in relapse rates between the two groups (Log Rank test *P*=0.827) (Figure 2A).

After 12, 24, and 36 months of treatment, surgical resection was performed in 7.8%, 15.0%, and 37.4% of patients on early immunomodulator therapy, and in 1.6%, 4.7%, and 31.8% of patients on conventional therapy, respectively, which was not a statistically significant difference (Log Rank test P=0.326) (Figure 2B).

There was no difference in the risk of disease-related hospitalization between the two groups at 12, 24, and 36 months, respectively (13.5%, 21.0%, and 50.0% vs. 1.1%, 6.8%, and 45.7%, Log Rank test *P*=0.228) (Figure 2C).

### Predictive factors related to disease relapse

According to univariate analysis, age, gender, body mass index (BMI), and disease activity as assessed by CDAI were not significant predictors of relapse (Table 2). In the analysis of ESR, CRP, and hematocrit, we selected cut-off points of 15, 8.0, and 40.4, respectively. Patients who had higher CRP levels relapsed more frequently compared with those who had lower CRP levels (odds ratio, 3.000; 95% confidence interval, 1.504–5.983; *P*< 0.005) (Table 2). However, there were no significant associations between disease relapse and ESR or hematocrit (Table 2).

**Table 2:** Univariate logistic regression analysis of baseline factors related to relapse

	**Valuables**	**OR (95% CI)**	***P*-value**
Age		0.999 (0.967-1.032)	0.940
Gender	Male		
	Female	0.769 (0.337-1.754)	0.533
BMI		0.980 (0.866-1.109)	0.748
CDAI severity	Mild		
	Moderate	1.957 (0.186-20.614)	0.576
	Severe	2.203 (0.222-21.849)	0.500
ESR*	> 15 mm/hr	0.745 (0.321-1.727)	0.492
CRP†	> 8.0 mg/L	3.000 (1.504-5.983)	0.002
Hct‡	> 40.4%	1.203 (0.622-2.329)	0.583

CDAI, Crohn’s disease activity index; BMI, body mass index; ESR, erythrocyte sedimentation rate; CRP, C-reactive protein; Hct, hematocrit; *cut-off value, 15; †cut-off value, 8.0; ‡cut-off value, 40.4.

### Adverse events and side effects

Eleven patients treated with early immunomodulator therapy experienced adverse events such as abdominal pain, bone marrow suppression, diarrhea, nausea, edema, and dermatitis (Table 3). Medication tolerance was very good overall in the patients on conventional therapy (Table 3). Early immunomodulator therapy was significantly associated with the development of an adverse event (*P* =0.029).

**Table 3:** Adverse events

	**Early immuno-suppressive therapy**	**Conventional therapy**	***P*-value**
Abdominal pain	1	–	
Bone marrow failure	4	–	
Diarrhea	–	1	
Nausea and vomiting	4	–	
Edema	1	–	
Dermatitis	1	–	
Total	11 (10.8)	1 (1.5)	0.029

## Discussion

For a considerable number of patients, the natural course of CD has a poor prognosis. Nearly all patients suffer from symptomatic flares and subsequent complications over a ten-year period [10]. Traditionally, symptom control was the principal goal of treatment. The conventional therapeutic strategy for CD dictates that medications are chosen based on the severity of symptoms.

In recent years, however, a change in the treatment goals for patients with CD has come under intense discussion. To maintain a good quality of life, and to keep the patient from suffering any irreversible consequences, very early intensive therapy (immunomodulators and/or biologics) has been suggested as a top-down treatment strategy [11]. The rationale for such an approach comes from rheumatology, where early intervention with immunomodulators or biologics is thought to prevent progressive destruction of joints [12].

There are few studies on the efficacy of early immunomodulator therapy in patients with CD, and they report many differences in clinical outcomes. These studies have shown that early administration of immunomodulators in patients with CD resulted in superior clinical outcomes when compared to conventional therapy, with a comparable safety profile [13]. On the contrary, in other studies, early immunomodulator therapy had no significant impact on CD course [2,14,15].

D'Haens G et al. found that early immunomodulator and biologic therapy was more effective than conventional management for induction of remission [5]. Likewise, we showed that early immunomodulator therapy demonstrated a higher rate of remission than conventional therapy, suggesting that early intervention with immunomodulators might have much more benefit in achieving clinical remission than the conventional strategy. Moreover, the steroid-free remission rate was also significantly higher in the early immunomodulator group. The maintenance of steroid-free remission is a major issue and one of the major treatment goals in these patients.

It is important to consider how long specific treatments maintain clinical remission when evaluating the effectiveness of a treatment. In a recent study, administration of immunomodulative agents within 6 months in CD patients was no more effective than conventional management in increasing the duration of clinical remission [14]. Similarly, in our study, there was no difference in the disease relapse rate between the two groups. There are some possible explanations for the above results. Most importantly, many of the patients might have been included in the early therapy group receive earlier immunomodulator therapy because of their poor clinical features at the start of treatment. Furthermore, a considerable number of patients with conventional therapy initiated treatment with immunomodulators as time passed in our study, which was also seen by D'Haens G et al. [5].

In choosing a treatment, we also should consider the adverse events and side effects. Early immunomodulator therapy showed a higher frequency of adverse events in this study. Because of these problems, it is important to identify the prognostic factors at the time of diagnosis, and earlier administration of immunomodulators should be recommended in selected CD patients with poor prognostic factors.

Surgical resection rates vary widely over time among published studies, ranging from 25 to 61% at 5 years in a recent review [16]. There are a few studies showing that early use of immunomodulators reduces surgical rates. For example, Lakatos et al. found that early immunomodulator use was associated with a significantly decreased time to first surgery in patients with CD [17]. However, we observed no significant difference in surgery rates between the two groups (Log Rank test *P* =0.326). There are some possible reasons for the differences in surgical resection rates between these studies. First, the diverse genetic predisposition of the subjects may be one of the major factors causing these differences [18]. Second, in the study by Lakatos et al., patients who had used biological agents were included in the early treatment group, which may also play a role. In Korea, biologics were launched into market very late, and patients are allowed to use biologics only after the failure of immunomodulators and corticosteroids.

The disease-related hospitalization rates observed in our study showed no difference between the two groups. Similarly, the AZTEC study investigated by Sans M et al. demonstrated that early use of immunomodulators did not reduce the disease-related hospitalization rate [19].

Several biomarkers have been evaluated as indicators of disease activity and predictors of the risk of relapse in patients with CD who are in remission [20]. CRP has been reported to be useful in predicting short-term prognosis and relapse [21-23]. In previous reports, a high baseline CRP was an independent predictor of relapse [23,24]. According to our results, patients with a baseline elevated CRP levels showed a higher relapse rate. Thus, this could play a role in predicting relapse at the start of treatment in newly diagnosed CD patients.

So far, nearly all studies on the efficacy of early immunomodulator therapy have focused on Caucasians. However, the clinical features of CD in Asian populations might be different from that in Caucasians [25]. Some studies investigating Asian populations suggest that the prognosis might be better in Asians compared to Western patients [26]. In this respect, this is the first study from an Asian population, which is one of the strengths of our study. Another strength of this study is that we demonstrate the efficacy and safety of early immunomodulator therapy in CD patients from a clinical practice setting. Patients encountered in real clinical practice often differ from those included in registrational trials. The former have a more heterogeneous mix of patients with co-morbidities and often have poorer treatment adherence than the latter. Thus, it is necessary to analyze the data from real clinical practice to confirm treatment efficacy.

The major limitation of our study is that it was a retrospective, observational study, which had the potential for selection bias and confounding factors. Another limitation comes from the small size of the studied population, which necessitates further prospective studies with a large number of patients.

## Conclusion

In conclusion, our study found that early immunomodulator therapy was more efficient than conventional therapy in terms of achieving clinical remission. However, early immunomodulator therapy failed to maintain clinical remission and adverse events were significantly more frequent. Given the findings that there was no difference in disease relapse, need for surgery, or hospitalization, and that adverse events were more frequent in early immunomodulator therapy, we cannot assert that early immunomodulator therapy is unequivocally better than conventional therapy. Therefore, we should evaluate whether patients have poor prognostic factors at baseline. Then, early immunomodulator therapy could be selectively applied.

## Competing interests

No potential conflict of interest relevant to this article was reported.

## Authors’ contributions

MSK and JHC contributed to the conception and design of the study. DHK, SJP, TIK, SPH, and WHK were responsible for acquisition, analysis and interpretation of data. MSK and JHC drafted the manuscript. All authors read and approved the final manuscript.

## Pre-publication history

The pre-publication history for this paper can be accessed here:

http://www.biomedcentral.com/1471-230X/14/85/prepub
